# An experience with the use of Misoprostol in second trimester miscarriages, in women with prior cesarean births

**DOI:** 10.12669/pjms.41.8.10884

**Published:** 2025-08

**Authors:** Nazia Liaqat, Qudsia Qazi, Fazeela Gul

**Affiliations:** 1 Dr. Nazia Liaqat, MBBS, FCPS (Obstetrics & Gynecology) Associate Professor, Department of Gynecology, Medical Teaching Institute, Lady Reading Hospital, Peshawar, Pakistan; 2 Dr. Qudsia Qazi, MBBS, FCPS (Obstetrics & Gynecology) Associate Professor, Department of Gynecology, Medical Teaching Institute, Lady Reading Hospital, Peshawar, Pakistan; 3 Dr. Fazeela Gul, MBBS FCPS II, Resident, Department of Gynecology, Medical Teaching Institute, Lady Reading Hospital, Peshawar, Pakistan

**Keywords:** Misoprostol, Missed miscarriage, Previous cesarean section

## Abstract

**Objective:**

To determine the success rate, frequency of complications and, side effects of misoprostol used, for second-trimester miscarriages, in women with history of previous cesarean section.

**Methodology:**

This cross sectional study was done at Lady Reading Hospital, Peshawar, from January 2021 to December 2022.The sample included 90 women, with a history of previous one cesarean section, undergoing medical management of second-trimester miscarriage. Outcomes noted were, success rate, frequency of scar complications and side effects.

**Results:**

The mean age of sample of 90 patients, was 28.87±4.6. The mean induction to expulsion time was 11 hours ± 3.9. Complete expulsion was achieved in 67.7% (61/90) cases. The need for surgical evacuation of retained products of conception, was observed in 32.5% (29/90) of cases. Scar complications were noted in 2.2% (2/90) cases. The need for surgical evacuation was more common with the vaginal route of administration as compared to the sublingual route i.e. 50% (20/40) vs 18% (09/50), p-0.001. Diarrhea was significantly associated with sublingual route of administration, p-0.001. Fever was common with the vaginal route, 47.5% (19/40) vs 26% (13/50), p-0.03. The mean induction to termination time was significantly shorter in the sublingual route group. (p-0.001)

**Conclusion:**

Misoprostol successfully and safely concludes the process of second trimester miscarriage in carefully selected women with a history of previous one cesarean birth.

## INTRODUCTION

With globally increasing rates of cesarean births, obstetricians are being increasingly faced with the challenges of dealing with pregnancy complications in the presence of scarred uterus**.** Management of second-trimester miscarriages and induced abortions in the presence of uterine scar are such significant issues. For decades now, misoprostol for inducing uterine contractions has been an efficient, safe and cost effective method of managing miscarriages and induced abortions.^1^ The induced miscarriage rate is estimated to be 29 per 1000 in women aged 15-44 years, with second trimester induced miscarriage accounting for 10-15% of all induced miscarriages, in a study.^2^ A success of more than 90% has been reported for combination of mifepristone and 400 ug of misoprostol, three hourly (maximum five doses) for second trimester abortions in women without uterine scar.^3^

There is global consensus on the safety and efficacy of misoprostol for managing second trimester miscarriages in women without a uterine scar. However, there are concerns about its safety for use in women with uterine scars. Surgical management in this population is associated with anesthesia complications, bleeding, infection, uterine perforation and scar complications. The evidence regarding safety and efficacy of various routes of misoprostol administration, is conflicting. Sublingual and vaginal route of administration were found to have no difference in success rate and side effects in one study.^4^ Another study reported superior efficacy for vaginal route in second trimester miscarriages, along with higher incidence of fever with vaginal route of administration.^5^

The present study was planned with the intent of finding out the success rate, rate of scar complications and various side effects of misoprostol for second trimester miscarriages, in women with uterine scar. This will reflect our experience of misoprostol use and will help our women choose a safe, effective and convenient method.

To determine success rate, frequency of uterine rupture, frequencies of side effects of misoprostol used for second trimester-miscarriages, in women with prior cesarean birth. Secondary objective was to determine impact of route of administration on these outcomes.

## METHODOLOGY

This cross-sectional study was done in the department of Obstetrics, Lady Reading Hospital Peshawar, Pakistan from January 2021 to December 2022.Ethical approval (Ref# 242/LRH/MTI; Dated: July 18, 2024) was obtained from hospital ethical approval committee. Sample size of 90 was calculated, assuming expected scar complication rate of 2% (factor of interest), the confidence level of 95% and margin of error of 4%.

### Inclusion Criteria:

- Women with history of previous one cesarean section, with second trimester miscarriage being medically managed with misoprostol 200 ug every three hourly protocol, were included in the study.

### Exclusion Criteria:

- Women with prior two or more cesarean deliveries, previous classical scar cesarean sections, multiple gestation, polyhydramnios were excluded. Those with other misoprostol regimen, mechanical induction, combined, or expectantly managed cases were also excluded.

Informed consents were taken from women before enrollment in to the study. Data regarding age, parity, period of gestation estimated from first/second trimester ultrasound, previous vaginal birth after cesarean delivery, BMI, route of misoprostol administration, induction to expulsion interval, need for surgical evacuation of retained products, occurrence of fever, nausea, vomiting, diarrhea, were recorded on a pre designed proforma. Post expulsion ultrasound was done to confirm completion of expulsion. Those who were found to have retained products were managed with manual vacuum aspiration. SPSS version 29.0 was used for statistical analysis. Qualitative variables were expressed as frequencies and percentages, and their associations were examined by either chi-square or Fisher’s Exact test. Quantitative variables were presented as mean and standard deviations and independent sample t -test was used to define associations. p value of <0.05 was determined to be significant.

## RESULTS

The mean age of sample of 90 patients, was 28.87±4.6, with the mean gestational age at the time of admission as, 16.41±3.1. Mean induction to expulsion time was 11 hours ± 3.9, (Table-I). In,78.8% (71/90) cases, the process initiated within the first 12 hours of first dose administration. Longer duration of time and more doses were needed in 21.1% (19/90) patients. Complete expulsion was achieved in 67.7% (61/90) cases. Sublingual route of administration was used in 55.6% (50/90) cases and vaginal route in 44.4% (40/90) cases. Surgical evacuation of retained products of conception, was needed in 32.5% (29/90). Scar complications were seen in 2.2% (2/90) cases. Vomiting was the commonest side effect, observed in 38.9% (35/90) cases, (Table-II).

**Table-I:** Demographic Features.

	N Statistic	Minimum Statistic	Maximum Statistic	Mean Statistic	Std. Deviation Statistic
Age of patient	90	19.00	40.00	28.8778	4.61514
Period of gestation	90	13.0	26.0	16.411	3.1591
Doses needed	90	1.0	7.0	3.511	1.2474
BMI of patient	89	24.0	33.0	28.169	2.3990
Induction to expulsion interval	90	4.0	22.0	11.067	3.9938
Duration of stay	90	1.0	4.0	1.244	.5260
Valid N (list-wise)	89				

**Table-II:** Demographic Features of Sample.

	Yes	No
Previous VBAC	38(42.2%)	52(57.8%)
Sublingual Route	50(55.6%)	40(44.4%)
Vaginal Route	40(44.4%)	50(55.6%)
Nausea and vomiting	35(38.9%)	55(61.1%)
Diarrhea	13(14.4%)	77(85.6%)
Fever	32(35.6%)	58(64.4%)
Scar complications	2(2.2%)	88(97.8%)

Successful completion of process, without the need for surgical intervention was more common with the sublingual route of administration. There was statistically significant association between need for surgical evacuation and route of administration of misoprostol, being more common with the vaginal route of administration as compared to the sublingual route i.e. 50% (20/40) vs 18% (09/50) respectively, p-0.001. Diarrhea was significantly associated with sublingual route of administration, p-0.001. Fever was common with the vaginal route, 47.5% (19/40) vs 26% (13/50), p-0.03. Scar complications were observed only with vaginal route, 5%, p-0.11, (Table-III). The mean induction to termination time was significantly shorter in the sublingual route group compared to vaginal, 8.66 hours vs 14.07, p-0.001, (Table-IV).

**Table-III:** Association of outcomes in relation to route of administration.

	Vaginal	Sublingual	p-value
Need for surgical evacuation	20(50%)	09(18%)	0.001
Nausea/Vomiting	19 (47.5%)	16 (32%)	0.13
Diarrhea	00	13 (26%)	0.001
Fever	19 (47.5%)	13 (26%)	0.03
Scar Complications	2 (5%)	00	0.11

**Table-IV:** Induction to miscarriage time in relation to route of administration.

	Mean Time in hours	Standard Deviation	Mean Difference	p-value
Vaginal route	14.07	3.2	5.4	0.001
Sublingual Route	8.66	2.7		

## DISCUSSION

The current study concluded that misoprostol successfully completes the process of termination in the majority of patients with previous uterine scar. In about two third of the cases, completion of miscarriage without further need for surgical intervention, is achieved. Scar complications with misoprostol are uncommon, in carefully selected cases with previous uterine scar. Sublingual route has better clinical profile in terms of reduced need for surgical evacuation, reduced incidence of fever, and shorter induction to expulsion interval.

In the present study overall, successful complete abortion was observed in 67.7% of women. A success rate of up to 60.5%(178/294) was observed with misoprostol used for first trimester termination in a study.^6^ Another study, reported complete miscarriage in 64 (67.3%) women with previous uterine scar and misoprostol treatment.^7^ One study reported success rate of 91.3% in 48 hours, in women with previous CS, using 400ug misoprostol intravaginally every six hours.^8^ These variations in the success rates of medical termination in cases of previous uterine scar can be attributed to heterogenicity in the study protocols for doses regimes, routes of administration, use of mifepristone, concurrent or subsequent use of oxytocin etc. The aim is to have an expeditious and complete termination and without any scar complications. In the current study half of the FIGO recommended dose for second trimester terminations, were used. Pre induction mifepristone was not used in the current study.

This is slightly higher than 0.9%, reported in a study involving women with uterine scar, undergoing second trimester abortion with misoprostol.^9^ In another retrospective study one out of 99 (1%) cases with previous one cesarean section developed scar complications with the use of misoprostol for second trimester miscarriages.^10^ In the same study there was no difference in the rates of scar complications between women with and without previous uterine scar. In many studies lower doses of misoprostol 100 or 200 microgram have been used. Different doses and regimen need to be compared in larger multicenter randomized controlled trials to have effective, safe and uniform dosing regimens. In a large systematic review, uterine rupture, was noted in 1.1% (10/874) women with prior cesarean birth as compared to 0.01% (2/6,244) in women without uterine scar.^11^ Similarly another study reported uterine rupture in 1.3% cases of medical abortions with misoprostol, in women with prior cesarean section. The same study also reported more complications like blood loss of more than 1000cc and placental retention in women with previous cesarean section compared to those without cesarean section.^12^ In a review of 164 studies carried out by Natalie and colleagues, it was found that incidence of uterine rupture after first trimester medical termination of pregnancy was 0.5% after previous one cesarean section and 2.2% after two or more cesarean section.^13^ Out of the two cases who experienced scar complications in the current study, one woman had hydropic baby and chorioamniotis. A case report from Turkey also reported uterine rupture with misoprostol in patient with chorioamniotis.^14^ In the literature, an increased incidence of uterine rupture -up to 6- folds- has been reported in women with a previous uterine scar and chorioamniotis, undergoing a trial of labour.^15^ Furthermore,the combination of labor augmentation/induction with chorioamniotis increases the risk of uterine rupture by up to 33- fold higher.

In the present study sublingual route was found to have a much quicker action, higher success rate in achieving complete expulsion. In a study done on patients with first trimester miscarriages, sublingual misoprostol was found to be more effective in achieving complete evacuation and reduced need for surgical intervention.^16^ In another study, sublingual administration was found to be more effective compared to vaginal administration in similar doses.^17^ In the present study the mean misoprostol administration to miscarriage time was 11.06±3.99 hours, this is shorter than 22.6±11.7, what was noted by Hatem and colleagues in their study.^18^ They had used smaller doses compared to present study, which could have led to longer time to termination in their study. In the current study nausea, vomiting and fever were more frequent with vaginal route of administration. Diarrhea was common in the sublingual group. A study reported gastrointestinal side effects and fever, more with oral administration compared to vaginal*.^19^*

In another study, different routes were noted to have same success rates. It was also reported that incidences of nausea, diarrhea and fever among different routes of administration, were not significantly different. Only vomiting was more frequently observed with sublingual administration.(p-0.03)^20^ and with all routes having same success rates as well. The dilemma of uniform, most effective and safe misoprostol regimen for use in women with prior uterine scar is still to be resolved. Until then, careful selection and close monitoring of these women under medical supervision is recommended to avoid complications. Women with second trimester terminations, in the presence of uterine scar present a unique challenge to the dealing obstetricians.

This study represents a single center experience in a highly selected group of patients with uniform doses and regimen of misoprostol use, for second trimester missed miscarriages only.

### Limitations

Limitations of the study are, lack of a control group and not addressing pre induction characteristics in the form of bishops scoring, vaginal bleeding and time from diagnosis of miscarriage to start of treatment. Patients with history of previous two or more cesarean sections were excluded from study as these patients are treated on case-to-case basis and their care is highly individualized.

## CONCLUSION

Misoprostol successfully and safely concludes the process of second trimester miscarriage in carefully selected women with a history of previous one cesarean birth.

### Author’s Contribution:

**NL:** Conceptualization, data analysis, drafting of manuscript.

**QQ:** Data collection, editing and finalization of manuscript.

**FG:** Data Analysis, literature search, manuscript editing.

**NL and QQ** are responsible for originality and integrity of the research.

## References

[1] Ak M, Dolanbay M, Kütük MS (2023). An analysis of misoprostol effectiveness in second trimester pregnancy terminations. J Surg Med.

[2] Sedgh G, Henshaw S, Singh S, Åhman E, Shah IH (2007). Induced abortion:estimated rates and trends worldwide. Lancet.

[3] Joensuu-Manninen H, Kuvaja P, Talvensaari-Mattila A (2010). Clinical efficacy of mifepristone and misoprostol in second trimester pregnancy termination. Acta Obstet Gynecol Scand.

[4] Milani F, Sharami SH, Arjmandi S (2014). Comparison of sublingual and vaginal misoprostol for second-trimester pregnancy terminations. J Fam Reprod Heal.

[5] Von Hertzen H, Piaggio G, Wojdyla D, Huong NTM, Marions L, Okoev G (2009). Comparison of vaginal and sublingual misoprostol for second trimester abortion:Randomized controlled equivalence trial. Hum Reprod.

[6] Nasser S, Makhdoom T, Alhubaishi LYA, Elbiss HM (2024). Medical management of first trimester missed miscarriages - A cross-sectional study. Pak J Med Sci.

[7] Mahmood Shakir H (2022). Safety of Vaginal Misoprostol for the Termination of Second Trimester Miscarriage in Women with Previous Uterine Scar in Iraq. Arch Razi Inst.

[8] Pongsatha S, Suntornlimsiri N, Tongsong T (2024). Comparing the outcomes of termination of second trimester pregnancy with a live fetus using intravaginal misoprostol between women with and without previous cesarean section. BMC Pregnancy Childbirth.

[9] Erturk A, Karapinar BT, Tasgoz FN, Dundar B, Kender Erturk N (2022). The safety of misoprostol alone use for second-trimester termination of pregnancy in women with previous caesarean deliveries. Eur J Contracept Reprod Heal Care.

[10] Bahar R, Alexandroni H, Karavani G, Gilad R, Benshushan A (2021). Safety of medical second trimester abortions for women with prior cesarean sections. Arch Gynecol Obstet.

[11] Henkel A, Miller HE, Zhang J, Lyell DJ, Shaw KA (2023). Prior Cesarean Birth and Risk of Uterine Rupture in Second-Trimester Medication Abortions Using Mifepristone and Misoprostol:A Systematic Review and Meta-analysis. Obstet Gynecol.

[12] Dickinson JE, Doherty DA (2023). Mifepristone priming and subsequent misoprostol for second trimester medical abortion in women with previous caesarean delivery. Aust New Zeal J Obstet Gynaecol.

[13] Drever N, Gangathimmaiah V, van Der Lugt B, O'Brien C, Melville C, Black K (2025). Induced Abortion After Previous Caesarean Section:A Scoping Review. Aust N Z J Obstet Gynaecol.

[14] Golshahi F, Yarandi F, Ramhormozian S, Shirali E (2022). Lessons learnt from cases of misoprostol-based pregnancy termination followed by uterine rupture:Report of 3 cases. J Obstet Gynecol Cancer Res.

[15] Vilchez G, Gill N, Dai J, Chelliah A, Jaramillo H, Sokol R (2015). 156:Rupture in the scarred uterus. Am J Obstet Gynecol.

[16] Rizwan A, Alam K, Iftikhar T, Farooq S (2022). Comparison of vaginal versus sublingual misoprostol in the treatment first trimester missed miscarriage. J Soc Obstet Gynaecol Pak.

[17] Boutalaq W, El Sayed Y, Behery M, Abdel Fatah M (2021). Administration of Misoprostol Sublingual versus Vaginal for the Termination of Second Trimester Missed Miscarriage. Zagazig Univ Med J.

[18] Hatem D, Hafez AA, Mostafa WAI (2013). Could vaginal misoprostol be effective in second-trimester pregnancy termination in women with a previous uterine scar. Evid Based Women's Heal J.

[19] Duong ML, Pham VL, Bui QN, Nguyen TH, Duong TKR, Ngo NH (2023). Evaluation of the Effectiveness of Oral and Vaginal Misoprostol for Intrauterine Fetal Death and Fetal Anomaly in Second Trimester Pregnancy Termination:a Randomized Clinical Trial. Tạp chíY Du?c học C?n Tho.

[20] Souizi B, Akrami R, Borzoee F, Sahebkar M (2020). Comparison of the efficacy of sublingual, oral, and vaginal administration of misoprostol in the medical treatment of missed abortion during first trimester of pregnancy:A randomized clinical trial study. J Res Med Sci.

